# Host-Derived Smooth Muscle Cells Accumulate in Cardiac Allografts: Role of Inflammation and Monocyte Chemoattractant Protein 1

**DOI:** 10.1371/journal.pone.0004187

**Published:** 2009-01-14

**Authors:** Piotr Religa, Monika K. Grudzinska, Krzysztof Bojakowski, Joanna Soin, Jerzy Nozynski, Michal Zakliczynski, Zbigniew Gaciong, Marian Zembala, Cecilia Söderberg-Nauclér

**Affiliations:** 1 Cellular and Molecular Immunology, Karolinska Institute, Stockholm, Sweden; 2 Department of Internal Medicine and Hypertension, Warsaw University of Medicine, Warsaw, Poland; 3 Department of General, Vascular and Oncologic Surgery, Warsaw University of Medicine, Warsaw, Poland; 4 Department of General Biochemistry and Nutrition, Warsaw University of Medicine, Warsaw, Poland; 5 Silesian Center for Heart Diseases, Zabrze, Poland; Uppsala University, Sweden

## Abstract

Transplant arteriosclerosis is characterized by inflammation and intimal thickening caused by accumulation of smooth muscle cells (SMCs) both from donor and recipient. We assessed the relationship between clinical factors and the presence of host-derived SMCs in 124 myocardial biopsies from 26 consecutive patients who received hearts from opposite-sex donors. Clinical and demographic information was obtained from the patients' medical records. Host-derived SMCs accounted for 3.35±2.3% of cells in arterioles (range, 0.08–12.51%). As shown by linear regression analysis, an increased number of SMCs was associated with rejection grade (mean, 1.41±1.03, p = 0.034) and the number of leukocytes (19.1±12.7 per 20 high-power fields, p = 0.01). The accumulation of host-derived SMCs was associated with an increased number of leukocytes in the allografts. In vitro, monocyte chemoattractant protein 1 (MCP-1) released from leukocytes was crucial for SMC migration. After heart allotransplantion, mice treated with MCP-1-specific antibodies had significantly fewer host-derived SMCs in the grafts than mice treated with isotypic antibody controls. We conclude that the number of host-derived SMCs in human cardiac allografts is associated with the rejection grade and that MCP-1 may play pivotal role in recruiting host-derived SMCs into cardiac allografts.

## Introduction

The major cause of late organ dysfunction after transplantation is vasculopathy characterized by vessel inflammation and intimal hyperplasia due to the recruitment of smooth muscle cells (SMCs) into the vessel intima [1], [2]. This process results in progressive luminal narrowing caused in part by a healing reaction in the intima. The intimal cells could be derived from phenotypically modulated medial SMCs within the graft or from host-derived SMCs [3]. Possible sources of the host-derived cells in cardiac allografts are cells in adjacent vessels that migrate toward the graft, circulating tissue progenitors, or possibly bone marrow–derived progenitors [4]–[6]. Although host-derived cells contribute to transplant vasculopathy, their clinical significance and the mechanisms of their accumulation in the intima are unknown.

Transplant vasculopathy is believed to have both immunological and nonimmunological causes and results in vascular dysfunction due to factors affecting the allograft [1]. Diverse immunological factors that contribute to chronic transplant dysfunction have been identified, including the degree of acute rejection, immunosuppression, and opportunistic infections, particularly cytomegalovirus infection [7], [8]. Nonimmunological factors, such as the age of the recipient, underlying diseases, and ischemia, also contribute to chronic transplant dysfunction.

In this study, we investigated clinical factors that influence the accumulation of host-derived cells in arterioles of human cardiac allografts and potential factors involved in their migration. We analyzed archived myocardial biopsies from heart transplant recipients mismatched in sex with their donors, which enabled us to determine the origin of SMCs in the vessel lesions. We also performed in vitro migration assays and in vivo heart transplantation studies in mice.

## Materials and Methods

### Biopsies of human cardiac allografts

We analyzed 124 post-transplantation cardiac biopsy specimens from 26 consecutive patients who received cardiac allografts from opposite-sex donors from 1994–2003. Specimens were from the tissue bank at the Silesian Center for Heart Disease (Zabrze, Poland). The protocol was approved by the regional board of the ethics committee at the Karolinska Institute and conformed to the principles outlined in the Declaration of Helsinki. All patients gave informed consent. Specimens were obtained by endomyocardial biopsy as part of a standard procedure for monitoring acute graft rejection (weekly for the first month, every 2 weeks for the second month, every 3 months until end of the first year, every 6 months during the second year, and yearly thereafter). Biopsies not containing arterioles were excluded from analysis.

Specimens were analyzed by pathologist using the criteria of the International Society for Heart and Lung Transplantation [9]. Rejection was graded according to the following scale: 0, no rejection; 1A, focal (perivascular or interstitial) infiltrate without necrosis; 1B, diffuse but sparse infiltrate without necrosis; 2, a single focus of aggressive infiltration and/or focal myocyte damage; 3A, multifocal aggressive infiltrates and/or myocyte damage; 3B diffuse inflammation and necrosis; and 4 diffuse aggressive polymorphous infiltrate, edema, hemorrhage, vasculitis, and necrosis. Samples were also analyzed by immunohistochemistry for the accumulation of host-derived SMCs in arterioles.

### Clinical information

Retrospective clinical and demographic data were collected from the patients' medical records. The clinical data included age, time from transplantation, underlying diseases (hypertension, diabetes, smoking, gastric ulcer, hepatopathy, episodes of thromboembolism, heart, lung and kidney failure, cancer, hypercholesterolemia), and blood morphology. Information about immunosuppression and infection with cytomegalovirus, hepatitis B virus, hepatitis C virus, and human immunodeficiency virus was obtained at the time of hospitalization for myocardial biopsy. To assess heart function, echocardiography was performed to estimate the ejection fraction.

### Immunohistochemistry

Immunohistochemistry was performed as described [10] with primary antibodies against human smooth muscle α-actin (αSMA), vonWillebrand factor (vWF), CD45, CD14, CD3, CD8, CD 4, IgG and IgM (Dako, Glostrup, Denmark), MCP-1 (Biolegend, San Diego, CA). Vessels positive for αSMA and vWF and cells positive for CD45, CD14, CD3, CD8, and CD4 were manually counted in 20 high-power fields (HPF) and averaged. IgG and IgM levels were scored as low, medium, or high.

### Laser capture microdissection of αSMA-positive arterioles

Laser capture microdissection was performed on tissue sections stained for αSMA. In three to four sections from each biopsy, 300–400 αSMA-positive cells were microdissected from arterioles with the PixCell II System (Arcturus Engineering, Mountain View, CA). The percentage of host-derived cells among captured cells was estimated by real-time PCR for the *SRY* gene as described [10], [11].

### In situ hybridization for chromosome Y

Tissue sections were rehydrated in xylene-graded ethanol, boiled in citrate buffer for 15 min to unmask epitopes, and incubated with anti-αSMA antibodies overnight at 4°C. After fixation in 4% formalin in phosphate-buffered saline for 15 min, the sections were placed in 2× saline sodium citrate (SSC)/0.5 NP-40 at 37°C for 30 min and dehydrated in 70% and 95% ethanol for 2 min each, denatured in 70% formamide/2× SSC at 72° for 20 min, and dehydrated in ice-cold ethanol. The sections were then hybridized with 10 µl of probe for 16 h in a humidified chamber and washed with 0.5× SSC for 5 min at 72°C, 50% formamide/2× SSC at 43°C for 15 min, and 0.1× SSC at 60°C for 15 min. Finally, the sections were counterstained with propidium iodide, mounted in Mountex (Vector), and examined by confocal microscopy (Leica TCS SP5).

### Cell migration assay

The migration of SMCs was measured with a modified Boyden chamber (NeuroProbe). Cells (60,000/well) were seeded into the upper chamber in serum-free Ham's F-12 medium containing 0.2% bovine serum albumin. Ham's F-12 medium containing monocyte chemoattractant protein (MCP) 1 (50 ng) or leukocyte-conditioned medium was added to the lower chamber. In migration inhibition experiments, we added MCP-1 antibodies to the medium in the lower chamber or anti-CCR2 to the cells 30 min before the experiment. After a 6-h incubation, the medium was removed, and cells attached to the bottom of the filter were fixed in 99% methanol, stained with a Giemsa solution, and examined by light microscopy. The mean number of cells per microscopic field (magnification, 20×) was plotted. All experiments were repeated three or four times.

### Mouse heart allograft transplantation

Balb mice were used as donors of vascularized cardiac allografts. The protocol was approved by the ethics committee at Warsaw Medical University and conformed to the principles in the Declaration of Helsinki. The mice were anesthetized with medetomidine (1 mg/g) and ketamine (75 mg/g), and vascularized cardiac grafts were collected and transplanted into recipient GFP mice as described [12]. The aorta and pulmonary artery of the donor heart were anastomosed to the recipient abdominal aorta and inferior vena cava, respectively. Allograft survival was evaluated by daily palpation. Cessation of beating was interpreted as rejection. Between days 5–10 after transplantation, mice received anti-MCP-1 or isotypic antibodies (10 mg/kg i.v.). The hearts were collected, cut into 5-µm-thick sections, stained immunohistochemically for CD45, CD68, CD3, CD8, CD4, and αSMA, and analyzed by confocal microscopy.

### Data presentation and statistical analysis

Data analyses and event classifications were performed by investigators blinded to the clinical information. Values are expressed as mean±SD or as means and medians with SD. Associations between accumulation of host-derived SMCs and predictors of cellular accumulation selected from demographical, biochemical/functional, and immunohistochemical data were analyzed by best-subset logistic regression. The Akaike information criterion and manual elimination of risk factors based on medical consideration were used to obtain a parsimonious model with good predictive capability. Leave-one-out cross-validation was used to avoid overfitting of the final model. The final model included only predictors with significant contribution (p<0.05). In all analyses, a two-sided p value<0.05 was considered significant. The results of studies in mice were analyzed with *t* tests; p<0.05 was considered significant.

## Results

### Patient characteristics

Myocardial biopsies were obtained from 26 consecutive patients who received cardiac transplants from donors of the opposite sex. The characteristics of the patients and the indications for transplantation are shown in Table 1. There were 15 women and 11 men (mean age, 41 years). The most common indications for transplantation were ischemic heart disease (n = 13) and congestive cardiomyopathy (n = 10). The patients were characterized by hypercholesterolemia (n = 16), smoking (n = 9), hypertension (n = 9), thrombotic episodes (n = 9), and diabetes (n = 5). All patients were treated with cyclosporin A, prednisone, and azathioprine/mycophenolate-mofetil.

**Table 1:** Baseline characteristic of the patients*.

Characteristics	Total	Females	Males
No. of patients	26	15	11
Number of biopsies (mean+range)	124	6.6 (4–14)	2.3 (1–4)
Mean age (years)	41±20.0	31.5±21.7	53.4±6.8
Treatment indication			
Congestive cardiomyopathy	10	5	5
Ischemic heart diseases	13	7	6
Hypertrophic cardiomiopaty	1	1	
Acquired valve disease	1	1	
Cogenital heart disease	1	1	
Diabetes	5	1	4
Smoking	9	4	5
Re-operation	2	0	2
Hypertension	9	4	5
Thromboembolic episodes	9	2	7
Hypercholesterolemia	16	8	8
Kidney failure	1	1	0
Gastric ulcer	2	2	0
Liver failure	2	0	2

* Values are numbers of patients, unless indicated otherwise.

### Histological findings

**Figure 1:**
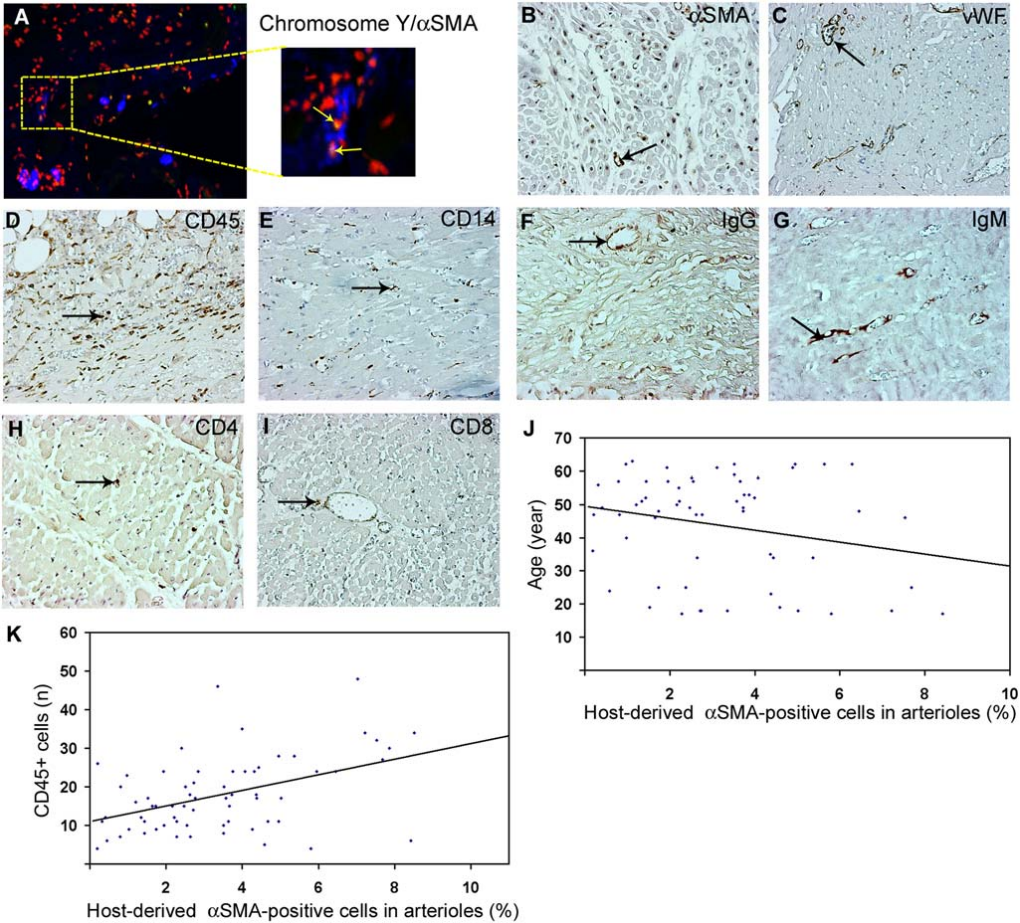
Immunohistochemical characteristic of human cardiac allografts. (A) Immunohistochemistry for αSMA (blue) followed by in situ hybridization for chromosome Y (green) and nuclear counterstaining (red). (B–K) Immunohistochemical staining of human cardiac allografts for αSMA (B), vWF (C), CD45 (D), CD14 (E), IgG (F), IgM (G), CD4 (H), and CD8 (I). Arrows indicate positive cells and staining. (J and K) Scatter plots showing the association between the number of αSMA-positive cells in the vessel and patient age (J) CD45+ cells (K).

To estimate the number of αSMA-positive host-derived cells in the graft, we used a real-time PCR assay for the *SRY* gene. In each biopsy, host-derived cells accounted for a mean of 3.4±2.3% (range, 0.08–12.5%) of αSMA-positive cells obtained by laser capture microdissection of arterioles. The presence of host-derived SMCs in the allografts was confirmed by in situ hybridization for chromosome Y and immunostaining for αSMA (Fig. 1A).

To estimate the extent of rejection, myocardial biopsy samples were graded for lymphocytic infiltration and necrotic changes as described [9]. Severe rejection (greater than grade 2) was found in only 29 (23%) samples. The numbers of arterioles was determined by staining for αSMA and vWF. During development of vasculopathy, signs of arteriolitis were visible by edema and detachment of endothelial cells, decreased staining for αSMA, luminal narrowing, and changes in morphological structure of vascular wall (Figure 1B, C). Leukocytes, macrophages, and lymphocytes were identified by staining for CD45, CD14, CD3, CD4, and CD8 (Fig. 1D–I). In all biopsies, the number of infiltrating cells was low but clearly increased with the rejection grade, as expected. CD45^+^ leukocytes were the predominant, which accumulated in perivascular regions in samples with grade 2 rejection and increased in number with increasing rejection grade. In grade 3 rejection, these cells also accumulated in areas of cardiomyocytes. In contrast, CD4^+^ and CD8^+^ lymphocytes were detected in perivascular areas and were rare in cardiomyocyte areas (Fig. 1H, I). As expected, IgG and IgM accumulated with increasing rejection grade (Fig. 1F, G).

### Identifying primary predictors of host-derived SMC accumulation in the arterioles

To identify factors associated with the accumulation of host-derived cells around arterioles in the grafts, we built three separate multiple logistic regression models based on demographic/clinical factors, cellular characteristic, and biochemical/functional variables (Table 2).

**Table 2:** Logistic linear regression analysis of factors associated with accumulation of vascular progenitor cells in arterioles.

Predicting factors	Mean	Median	SD	Lower quartile 25%	Higher quartile 75%	p
Demographic						
Time from biopsy (mo)	12.238	9	12.472	2	18	0.054
Age (year)	35.990	40	20.008	17.25	52.75	0.010
Rejection grade	1.414	1	1.031	1	2	0.010
Immunohistochemical						
αSMA^+^ vessels (n)	7.200	7	3.813	5	9	0.013
vWF^+^ vessels (n)	14.206	10	10.280	7	17	0.010
CD45^+^ cells (n)	19.057	17	12.665	11	24	0.035
CD14^+^ cells (n)	12.500	7	15.162	4	16	0.209
CD3^+^ cells (n)	16.431	11	13.379	6	25	0.048
CD8^+^ cells (n)	5.073	4	4.031	2	7	0.382
CD4^+^ cells (n)	7.269	6	4.370	4	10	0.897
Biochemical/functional						
Ejection fraction (%)	57.809	57	6.470	53	64	0.445
Hematocrit (%)	33.453	33	3.826	31	36	0.612
Hemoglobin (g/dl)	7.322	7.3	1.022	6.7	7.7	0.035
White blood cells (×10^6^/l)	7.013	6.45	2.889	4.90	8.22	0.884
Cholesterol (mmol/l)	5.487	5.2	1.909	4.470	6.20	0.724
Triglycerides (mmol/l)	7.027	1.7	43.462	1.180	2.40	0.766
HDL (mmol/l)	2.037	1.5	4.536	1.1900	1.6350	0.645
LDL (mmol/l)	2.966	2.9	0.878	2.20	3.55	0.679
Cyclosporin A (ng/ml)	293.796	265.9	149.682	194.00	374.50	0.010

HDL, high density lipoprotein; LDL, low density lipoprotein.

#### Demographic and clinical factors

This model included time from biopsy, age, gender, rejection grade, and coexisting diseases (Table 1). The presence of host-derived SMCs in the allografts (Table 2, Fig. 1A) correlated significantly with mean age (40.0±20.0 years, p = 0.01) (Fig. 1J) and mean rejection grade (1.4±1.03, p = 0.01). However, several categorical risk factors, including coexisting diseases and smoking habits, were excluded from the model because of the low number of patients. Biopsy samples were obtained 1–70 months (mean, 12.2 months) after transplantation.

#### Cellular characteristics

In a best-subset logistic regression analysis, the number of arterioles staining positively for αSMA and vWF, the number of CD45^+^ leukocytes, and rejection grade independently predicted the accumulation of host-derived SMCs in the allografts. SMCs were significantly less abundant in areas with higher numbers of vWF-positive vessels (mean 14.2±12.3, p = 0.013) and αSMA (mean 7.2±3.8, p = 0.01). The number of host-derived cells increased with the number of CD45^+^ leukocytes (19.1±12.7, p = 0.035) (Fig. 1K). In a step model, those factors were the best predictors of increased accumulation of host SMCs in arterioles (Table 2).

#### Biochemical and functional parameters

This regression model included blood morphology, lipid data (e.g., plasma levels of total cholesterol, high density lipoproteins, and low density lipoproteins), and plasma concentration of cyclosporin A. Among functional tests of the heart, only the ejection fraction was included; data on other parameters were not collected at the time of biopsy. Hemoglobin and cyclosporin A were associated (p<0.05) with the accumulation of host-derived SMCs in the graft (Table 2).

### Final logistic regression model

The final model, based on results from the partial models, included the rejection grade, the number of arterioles and CD45^+^ leukocytes, hemoglobin levels, and plasma cyclosporin A levels (Table 3). The number of arterioles was significantly related (p<0.05) to the number of accumulated SMCs. The number of host-derived SMCs in arterioles was increased and associated with the rejection grade (mean 1.41±1.03, p = 0.034) and the number of leukocytes (mean 19.06±12.66 per 20 HPF, p = 0.01). Cyclosporin A levels correlated inversely with the accumulation of host-derived SMCs (p = 0.14).

**Table 3:** Logistic linear regression analysis of the association between predicting factors and accumulation of vascular progenitor cells in arterioles.

Predicting factor	Mean	Median	SD	p
Rejection grade	1.41	1	1.03	0.034
αSMA^+^ vessels (n)	7.20	7	3.81	0.041
CD45^+^ cells (n)	19.06	17	12.66	0.010
Hemoglobin (g/dl)	7.32	7.3	1.02	0.279
Cyclosporin A (ng/ml)	293.78	265.9	149.68	0.143

### MCP-1 is a crucial factor for migration of SMCs

**Figure 2:**
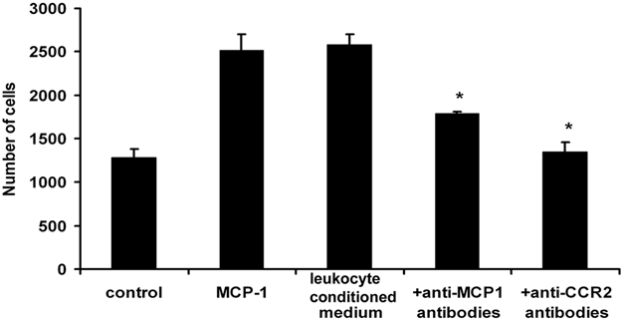
Migration of SMCs *in vitro*. SMC migration was induced by leukocyte-conditioned medium and MCP-1 and inhibited by neutralizing antibodies against MCP-1 and CCR2. *p<0.05.

Since the number of leukocytes was associated with increased accumulation of host-derived SMCs in human cardiac allografts, we determined whether leukocytes can influence migration of SMCs. In response to stimulation with leukocyte-conditioned medium, SMC migration increased by approximately 90% (Fig. 2). We recently observed that RNA levels of MCP-1, RANTES, and IP10 are highly increased in aortic allografts. Since MCP-1 is the major factor for monocyte recruitment to inflamed tissues, we hypothesized that it might also be involved in the migration of host-derived SMCs. Indeed, in response to recombinant MCP-1 (50 ng/m), SMC migration increased to levels similar to those induced by leukocyte-conditioned medium. SMC migration was inhibited by anti-MCP-1 and anti-CCR2 antibodies added to leukocyte-conditioned medium (Fig. 2).

**Figure 3:**
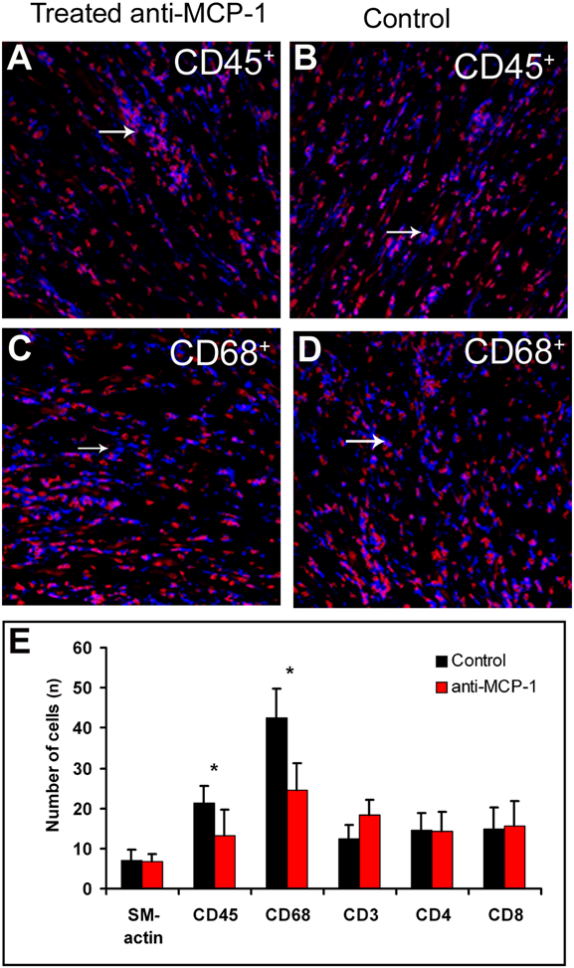
Immnunohistochemical analysis of mouse cardiac allograft treated anti-MCP-1 or isotypic control. (A and B) Distribution of CD45^+^ leukocytes in cardiac allografts treated with anti-MCP-1 antibodies (A) and control isotypic antibodies (B). (C and D) Distribution of CD68^+^ leukocytes in cardiac allografts treated with antibodies against MCP-1 antibodies (C) and control isotypic antibodies (D). Blue, positive signal; red, nuclear counterstaining. (E) Numbers of αSMA-positive vessels and leukocytes expressing CD45, CD68, CD3, CD4, and CD8. *p<0.05.

**Figure 4:**
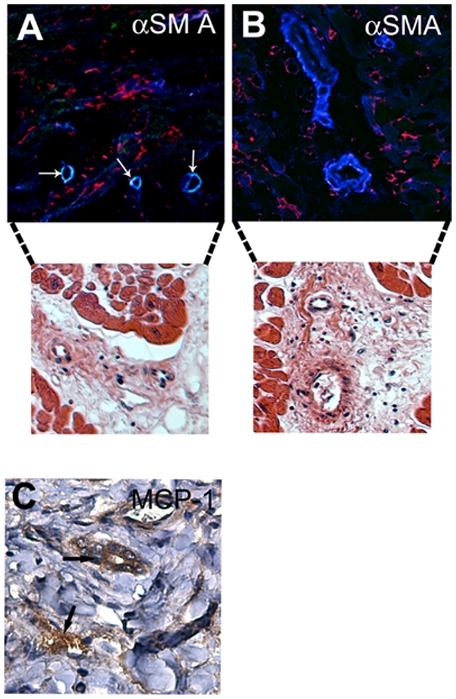
Staining of mouse cardiac allografts for αSMA to identify the origin of cells expressing αSMA. (A and B) Host-derived SMCs were present in arterioles with a single layer of SMCs (yellow) (A) but not in those with more SMC layers (B). Arrows indicate host-derived cells. Blue, αSMA; green, green fluorescent protein; red, nuclear counterstaining. Confocal microscopy analysis is followed by hematoxylin-eosin staining of parallel sections in order to present structure of vessels. Staining of human cardiac allografts for MCP-1 revealed MCP-1 around the small arterioles in an area with inflammation (C).

To further examine the role of MCP-1 in SMC recruitment to allografts, we transplanted hearts from Balb mice into B56/c57-GFP mice. Recipients were treated with anti-MCP-1 antibodies or isotype control antibodies for 5 days, starting on day 5 after transplantation. At sacrifice on day 10, the number of host-derived SMCs and the grade of the inflammatory response in the graft were assessed by confocal microscopy. Macroscopically, the control hearts appeared to be more edematous. Microscopic analysis revealed that anti-MCP-1 treatment significantly decreased the number of CD45^+^ and CD68^+^ leukocytes (Fig. 3A–E), suggesting a crucial role for MCP-1 in the inflammatory response. We did not observe a difference in CD3^+^, CD8^+^, or CD4^+^ lymphocytes. Owing to the lower sensitivity of the CD3-specific antibodies, the number of CD3^+^ cells was less than the sum of CD4^+^ and CD8^+^ cells. Host-derived cells were mostly present in small arterioles and were scarce in large arterioles (Fig. 4A, B). MCP-1 treatment also decreased the number of host-derived SMC in arterioles (1.58±0.53% vs. 0.89±0.68% in controls, p<0.05). Consistent with these findings, immunostaining for MCP-1 in human cardiac allografts showed that MCP-1 was expressed to a much greater extent around small arterioles and in areas with increased cellular inflammation (Fig. 4C). The results further imply that MCP-1 plays a pivotal role in the accumulation of host-derived SMCs in cardiac allografts.

## Discussion

In this study, we analyzed the accumulation of host-derived αSMA-positive cells in arterioles in biopsies of human cardiac allografts. Regression analysis of the clinical data indicated that the number of host-derived SMCs in arterioles was associated with acute rejection and the number of infiltrated leukocytes in the allografts. In vitro, MCP-1 released from leukocytes was crucial for SMC migration. In mice that received allogenic heart transplants, MCP-1 was a major factor in SMC recruitment to transplanted vessels; mice treated with anti-MCP-1 antibodies had significantly fewer host-derived SMCs in their grafts. These observations suggest that inflammation and MCP-1 are pivotal in the recruitment of host-derived SMCs into transplanted organs.

During acute rejection episodes, transplanted grafts exhibit different grades of inflammation, which lead to apoptotic and necrotic tissue damage in parallel with the initiation of healing. During inflammation, leukocytes release proinflammatory cytokines (e.g., interleukins 1α and 6, tumor necrosis factor-α, MCP-1, stromal cell–derived factor 1, and transforming growth factor β1) that contribute both to initiation of the inflammation and to healing [13]. We found signs of acute inflammation and an increase in the number of host-derived SMC that parallel the grade of rejection. These findings suggest that inflammation is a factor in the recruitment of SMCs into transplanted grafts.

Acute rejection has been directly related to cardiac death and to transplant vasculopathy characterized by occlusive vascular narrowing due to accumulation and proliferation of SMCs in the vascular intima, leading to intimal hyperplasia [14]. Previously, we showed that host-derived SMCs appear in the graft early after transplantation and start to accumulate within 1 month [10]. Since the number of SMCs was not related to the time between transplantation and biopsy, we hypothesized that SMC recruitment is facilitated by other factors, such as immune-mediated damage of the heart after transplantation [15].

Indeed, the number of accumulated host-derived cells in the graft correlated strongly with the number of CD45^+^ leukocytes and the grade of rejection, confirming that inflammation is strongly related to the recruitment of host-derived cells into the graft vessels. In support of this hypothesis, we previously found that an allogenic response in rats leads to apoptosis of transplanted SMCs that may affect the survival of host-derived cells that migrate to the transplanted vessels [10]. We have also shown that an immunosuppression regimen can protect the allograft from damage and reduce the recruitment of host-derived cells [16]. Transplant recipients with lower rejection grades or no acute rejection episodes have a lower risk of late chronic rejection and in particular transplant vascular sclerosis [17]. Thus, inflammation may be an underestimated cause for increased recruitment of progenitor cells to injured tissue to initiate vascular repair.

In studies to identify the inflammatory factors involved in the migration of SMCs in vitro, we found that SMCs migrated in response to leukocyte-conditioned medium and that MCP-1 and its receptor CCR2 were major factors in that migration. In vivo, MCP-1 and CCR2 played a pivotal role in recruiting SMCs into heterotopic heart grafts in mice. These observations support a crucial role for MCP-1 and CCR2 in the recruitment of vascular progenitor cells, perhaps to assist in healing. Indeed, MCP-1 is thought to contribute to healing of vascular injury [18] by recruiting vascular progenitor cells [19]. In atherosclerosis, MCP-1 is involved in recruitment of monocytes/macrophages into the vascular wall and in the formation of lipid cores and atherosclerotic plaques [20].

Other nonspecific risk factors for transplant vasculopathy, including smoking, hypertension, and coronary artery disease, did not correlate significantly with the number of host-derived SMCs in the vascular wall, perhaps because of the small numbers of patients with those risk factors. Reduction of circulating endothelial progenitor cells in the blood seems to be related to the level of vascular damage [21]–[24]. Moreover, factors such as hypertension and diabetes appear to impair the migration of endothelial progenitor cells [24]. Vascular progenitor cells appear to be specifically recruited into injured tissues, but the factors leading to their recruitment and their functions in the healing of tissue damage are poorly understood [25], [26]. The physiological status of arteries in these diseases affects the number of circulating progenitors by influencing their maturation, release from the bone marrow, and accumulation in injured tissues [27]–[29]. Thus, inflammation may lead to the recruitment of these cells. However, our findings do not indicate the source of host-derived SMCs and vascular progenitors.

In summary, we provide evidence that inflammation and MCP-1 are pivotal in the recruitment of host-derived SMCs into transplanted hearts. This knowledge may be useful in designing protocols aimed at reducing the number of host-derived SMC in cardiac allografts and increasing the number of progenitor cells to limit tissue damage and facilitate healing at sites of tissue injury.
